# Non-communicable diseases prevention and control in Pakistan: recommendations from policy and public health experts

**DOI:** 10.1186/s12919-025-00350-4

**Published:** 2025-10-14

**Authors:** Samina Akhtar, Namra Aziz, Farhala Baloch, Zeerak Jarrar, Shahid Khan, Sawera Hanif, Sana Qamar, Muhammad Imran, Maria Khattak, Safia Awan, Aysha Almas, Ayeesha Kamal, Zainab Samad

**Affiliations:** 1 Aga Khan University, Stadium Road, PO Box 3500, Karachi, Pakistan; 2 Khyber Medical University, Peshawar, Pakistan

**Keywords:** Non-communicable diseases, Policy recommendation, Panel discussion, Cardiovascular diseases, Mental health, Primary prevention

## Abstract

**Background:**

Non-communicable diseases (NCD) represent a significant and growing global health challenge, disproportionately affecting low- and middle-income countries (LMICs) like Pakistan. Despite their profound public health and economic implications, efforts to address NCD remain fragmented and insufficient. Collaborative platforms play a crucial role in fostering innovation and shaping policies to tackle this crisis effectively. Addressing the significant gaps in NCD initiatives and interventions in LMICs, particularly in Pakistan, the Aga Khan University (AKU), organized a one-day symposium, *AKUPI NCDs Research Symposium: A Dialogue on NCDs*. This symposium convened policy makers and public health experts from both local and international institutions. This paper synthesizes expert recommendations from a national symposium designed to identify actionable strategies for NCD prevention and control in Pakistan.

**Methods:**

Five expert panel discussions were conducted on themes critical for Pakistan: cardiovascular health, cancer prevention, mental health, economic perspectives, and sustainable urban design. The discussions were transcribed and analyzed using Braun and Clarke's thematic analysis framework.

**Findings:**

The analysis of discussions from over 30 national and international experts yielded 23 distinct themes. Key recommendations included: 1) Legislating a National NCD Act to establish dedicated units; 2) Shifting resources from tertiary to primary prevention, including integrating CVD risk assessment into existing Lady Health Worker programs; 3) Implementing task-shifting for mental health first aid; 4) Launching targeted, community-co-designed anti-stigma campaigns; and 5) Mandating sustainable urban design principles like the '3–30-300' rule. A critical gap was the absence of dedicated NCD units within the health system and a national policy for NCD and mental health.

**Conclusion:**

The symposium achieved a multi-sectoral expert consensus on a prioritized agenda. These insights provide a clear roadmap for policymakers, emphasizing that effective NCD control requires moving beyond siloed healthcare interventions to address broader social, economic, and environmental determinants through concrete, context-specific policies.

**Supplementary Information:**

The online version contains supplementary material available at 10.1186/s12919-025-00350-4.

## Background

The epidemic of non-communicable diseases (NCD), exacerbated by globalization and urbanization, poses a challenge, particularly for low- and middle-income countries (LMICs), where 85% of premature deaths are NCD-related. According to the United Nations Development Program's findings, there is a 60% likelihood of dying from major NCD between the ages of 30 and 69 in LMICs, compared with 10% in developed nations [1, 2]. LMICs are falling far behind in achieving the Sustainable Development Goals (SDGs) for health for all by 2030 [3].

The economic impact of NCD is also profound, affecting individuals and families by limiting employment opportunities, reducing income, and increasing healthcare costs, further hindering the efforts to achieve SDGs and making it crucial to integrate health policies with economic and social policies to effectively tackle the multifaceted challenges posed by NCD [4]. It also highlights the urgent need for concerted efforts to address the NCD crisis and international cooperation to mitigate the social and economic impacts of these diseases.

Acknowledging the significant gaps in NCD initiatives and interventions in LMICs, particularly in Pakistan, the Aga Khan University (AKU) in Karachi organized a one-day symposium on 4th December 2023, *AKUPI NCDs Research Symposium: A Dialogue on NCDs*. This event brought together policymakers and public health experts from local and international institutions, creating a platform to identify key policy and implementation gaps and establish priority areas for future research and generate actionable recommendations for NCD prevention and control.

The aim of this paper is to analyze these expert discussions and synthesize consensus-based priority recommendations for advancing NCD prevention and control in Pakistan.

## Methods

### Design and context

**Fig. 1:**
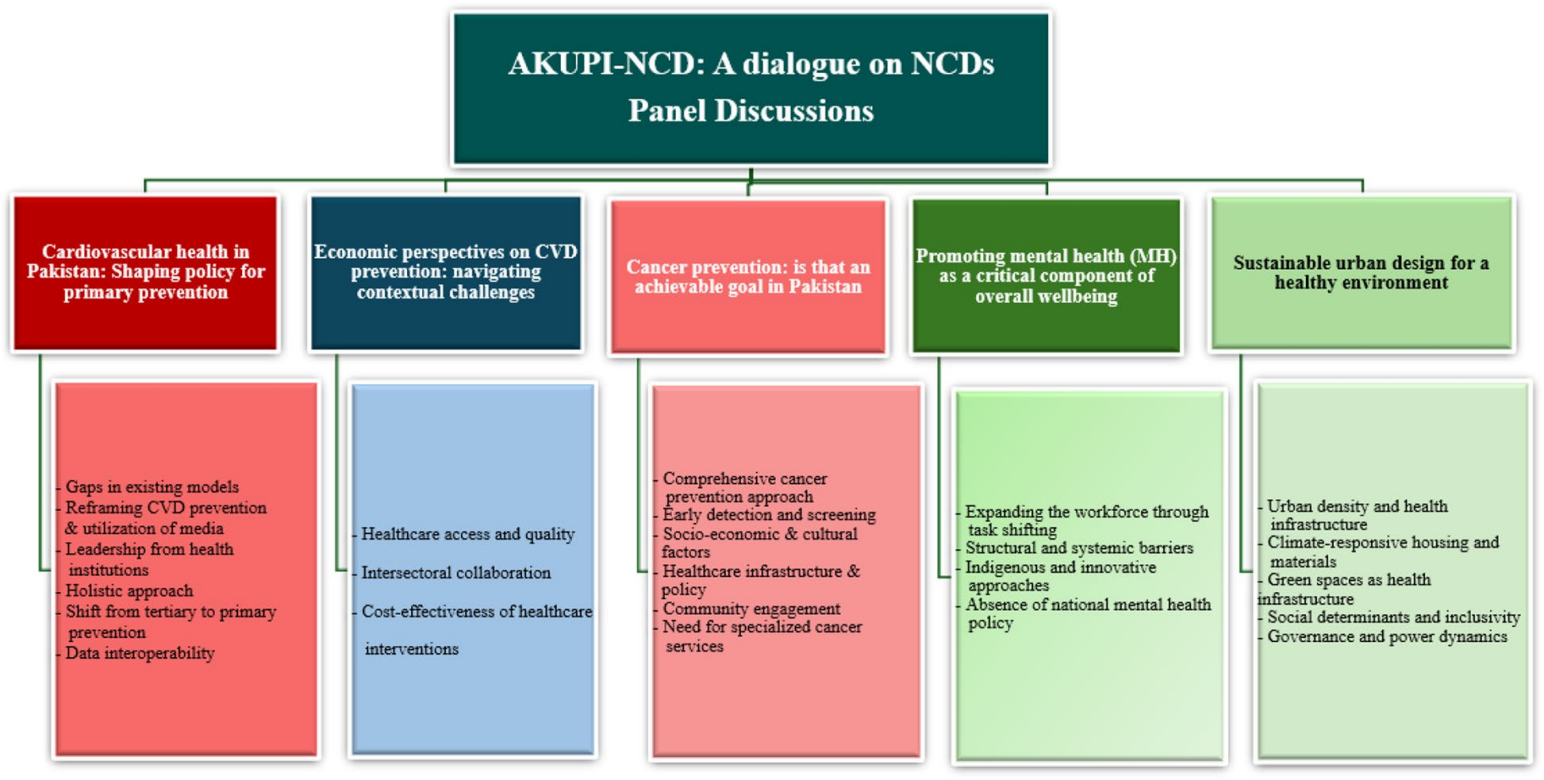
Overview of the panel discussion topics and key themes

The symposium was structured around panel discussions focused on five key NCD areas (Fig. 1). These areas were selected by the organizing committee based on (i) their alignment with the WHO NCD Global Action Plan priorities; (ii) the high disease burden of cardiovascular diseases (CVD) and cancer in Pakistan; (iii) the significant treatment gap in mental health; and (iv) the recognized impact of economic and environmental determinants on NCDs. Each panel discussion sessions lasted for 45 min, followed by a 15-min question-and-answer segment. All discussions were conducted in English and were audio- and video-recorded by the Audiovisual Library of AKU. Approximately 300 participants attended the symposium. The thematic analysis was conducted exclusively on the contributions of the panel experts. Attendees included healthcare professionals, public health researchers, policymakers, academics, educators, innovators, entrepreneurs, and students from Pakistan and abroad.

### Data analysis

The audio-visual recordings of the panel discussions were transcribed verbatim and analyzed using Braun and Clarke’s (2006) six-step framework for thematic analysis [5]. This framework involves: (1) familiarization with the data, (2) generating initial codes, (3) searching for themes, (4) reviewing themes, (5) defining and naming themes, and (6) producing the report.

First, researchers (SA, NA, FB, ZJ, SK) read and re-read the transcripts to immerse themselves in the data. They independently generated initial codes manually for a portion of the transcripts. The team then met to compare codes, develop a preliminary codebook, and resolve discrepancies through discussion to ensure consistency. This consensus-based codebook was then applied to the remaining data. The broader categories and preliminary themes that emerged from the codes were iteratively refined through discussions. The themes were then reviewed and finalized to ensure internal coherence and accurate representation of the data.

To maintain reflexivity, researchers kept personal journals to bracket their own assumptions and prior experiences in NCD research. Regular peer debriefings within the analysis team were conducted to challenge interpretations, ensure transparency, and strengthen the credibility of the findings.

## Findings

**Fig. 2:**
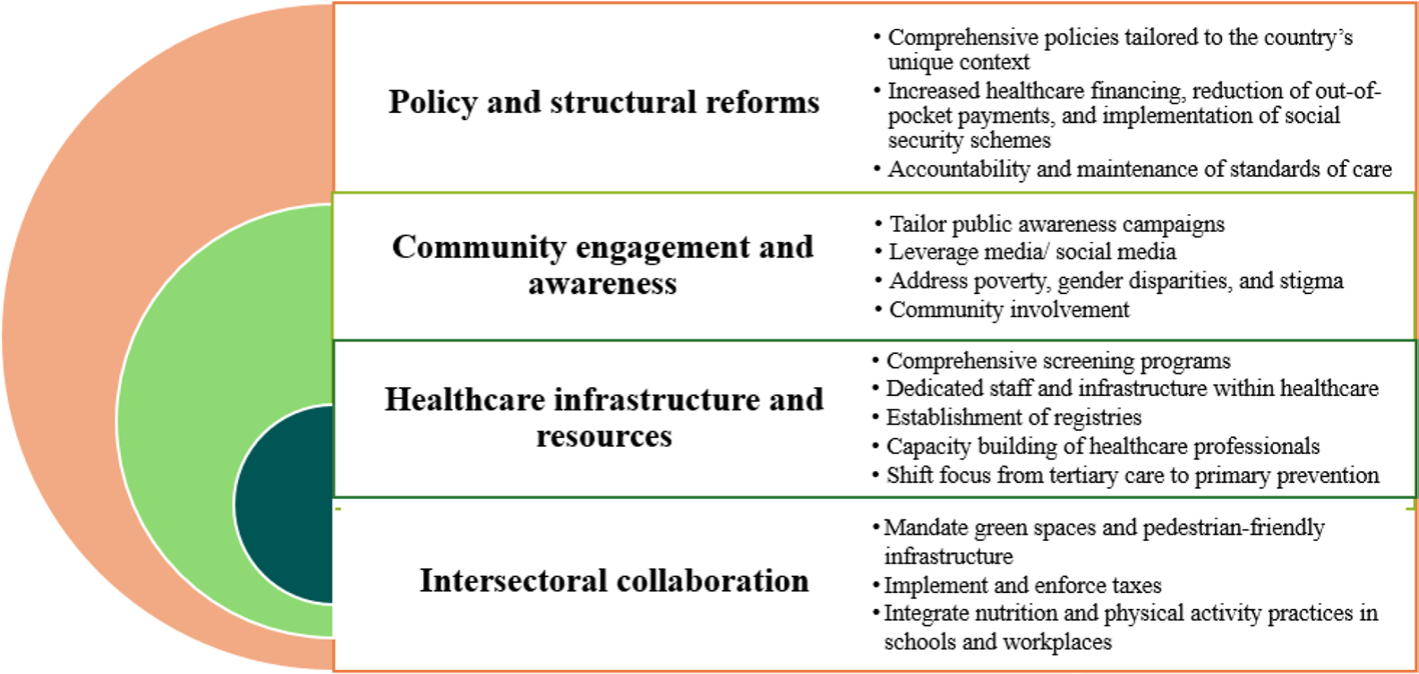
Recommendations for NCD prevention and management identified across five panel discussions

Analysis of the five panel discussions yielded 23 key themes. Four powerful cross-cutting themes emerged, highlighting systemic issues within Pakistan's approach to NCD: (1) severe fragmentation and under-resourcing of NCD services, particularly at the primary care level; (2) significant financial barriers for patients and underfunded public health initiatives; (3) a critical need for multi-sectoral collaboration beyond the health sector; and (4) the role of socio-cultural factors and stigma in hindering care-seeking behaviors. The following sections present the themes within each panel. Direct quotes from experts are provided in the Supplementary file. Overall recommendations are summarized in Fig. 2, with detailed panel-specific recommendations in Table 1.

**Table 1:** Panel-specific recommendations to address key NCD issues in Pakistan

Key Recommendations	Target Audience
1. Cardiovascular Health in Pakistan: Shaping Policy for Primary Prevention	
- Pass a National NCD Act to mandate the establishment of dedicated NCD units - Collaborate with research institutions to translate evidence into practice - Mandate the integration of CVD risk assessment into existing Lady Health Worker (LHW) and primary care protocols - Include CVD prevention in Maternal and Child Health Programs - Empower NCD Focal Persons at federal and provincial levels with a clear mandate for intersectoral collaboration	Ministry of National Health Services, Regulation & Coordination (NHSRC), Provincial Health Departments, Primary Healthcare Directorate, Planning Commission Pakistan, Media Regulatory Authorities
2. Economic Perspectives on CVD Prevention: Navigating Contextual Challenges	
- Legislate a mandatory increase in the health budget Develop a public dashboard to track the availability of essential NCD medicines, staffing levels, and quality of care at district levels - Fund and publish cost-effectiveness analyses of preventive interventions to build a compelling case for sustained investment - Develop a transition plan to reallocate health budgets from tertiary care infrastructure to primary care and prevention	Ministry of Finance, Federal Board of Revenue (FBR), Planning Commission, Provincial Finance Departments NHSRC, Educational Institutions
3. Cancer Prevention: Is That an Achievable Goal in Pakistan?	
- Formulate and resource a National Cancer Control Plan with specific, measurable targets for prevention, early detection, and treatment - Establish a National Cancer Registry with mandatory reporting from all public and private oncology centers to guide resource allocation - Integrate evidence-based screening for breast and cervical cancer into primary care centers - Launch targeted campaigns co-designed with communities to reduce stigma and improve health-seeking behavior, utilizing trusted local leaders and influencers	NHSRC, Provincial Health Departments, Pakistan Atomic Energy Commission (PAEC) Communications Agencies; Local Government & Community Development Departments
4. Promoting Mental Health as a Critical Component of Wellbeing	
- Pass updated legislation with a clear implementation plan and dedicated budget - Mandate and fund a national training program to equip GPs and LHWs with skills for identifying, managing, and referring common mental disorders - Launch a national anti-stigma campaign targeting the general public and healthcare providers, funded by the Ministry of Information	NHSRC, Provincial Health Departments; Pakistan Medical & Dental Council (PMDC) Ministry of Information & Broadcasting
5. Sustainable Urban Design for a Healthy Environment	
- Mandate the 3–30–300 rule (3 trees visible, 30% tree canopy, green space within 300 m) for all new housing developments and public buildings - Offer tax rebates or density bonuses to developers who incorporate certified green ventilation, insulation, and accessible green spaces - Require developers and city authorities to conduct inclusive consultations with residents from diverse social strata before approving major projects - Reform zoning laws to create walkable neighborhoods where schools, parks, and basic shops are within a 500-m walk for residents	Ministry of Climate Change, Provincial Housing & Urban Development Departments, all City Development Authorities (CDA, LDA, KDA, etc.), Pakistan Environmental Protection Agency, Chamber of Architects & Planners

### Cardiovascular health in Pakistan: shaping policy for primary prevention

CVD are on the rise in Pakistan, according to the 2019 Global Burden of Disease study, the estimated age-standardized incidence of CVD in Pakistan was 918.2, and the age-standardized death rate was 357.9 per 100,000 [6]. The objectives of this panel discussion were to explore successful models for CVD prevention and the role of policymakers in promoting primary prevention of CVD. The major themes emerged from the discussion were:

#### Gaps in existing models

Panelists agreed that despite the high prevalence of CVD, Pakistan lacks successful, scalable prevention models. Infrastructure gaps, low awareness in rural areas, and the absence of dedicated staff and offices for CVD within health departments were identified as major barriers. While vertical programs exist for immunization and communicable diseases, no equivalent structures exist for CVD/NCD. In urban settings, the absence of strong policies and mechanisms for implementation limits impact. Panelists emphasized the need for empowered leadership at the federal and provincial levels to design national programs for CVD prevention.

#### Reframing CVD prevention and utilization of media

Prevention should be framed as *health promotion* rather than disease prevention. Panelists suggested that messaging focused on well-being and quality of life resonates more with the general population, particularly young and middle-aged adults. Media and social media platforms were seen as critical tools for shifting narratives and engaging communities in proactive health behaviors. Policymakers and health professionals should adapt language, delivery, and framing the message to the needs, preferences, and cultural realities of different target groups, avoiding one-size-fits-all approaches.

#### Leadership from health institutions

Health institutions were identified as key drivers of policy advocacy. Collaboration between research institutions and policymakers is crucial for translating evidence into practice. Early and sustained dialogue can help researchers align their work with policymaker' priorities and improve policy uptake.

#### Holistic approach: a continuum of care starting in utero

Integrating CVD prevention efforts with ‘Maternal and Child Health Programs was suggested as a strategic approach to address the early determinants of NCD and promote preventive interventions from an early age, to address the multifaceted determinants of cardiovascular health.

#### Shift from tertiary to primary prevention

Participants highlighted the imbalance in Pakistan’s health investment, where tertiary hospitals receive disproportionate funding compared with primary health centers. Shifting resources toward primary prevention and community-level health promotion was recommended. Strengthening basic health units, rural health centers, and community health workers (e.g., Lady Health Workers and midwives) was seen as a cost-effective way to reduce the national CVD burden.

#### Data interoperability: essential for continuity of care

Panelists noted that despite years of patient data collection, the absence of interoperability across health platforms remains a major barrier. Patient records often fail to transfer between primary and tertiary care, leading to repeated histories and delayed treatment. Establishing interoperable health information systems was described as essential for continuity of care and improved outcomes.

### Economic perspectives on CVD prevention, navigating contextual challenges

In Pakistan, CVD imposes a significant economic burden across various dimensions of society. Against this backdrop, the objectives of this panel discussion were to elucidate the direct and indirect costs stemming from the burden of NCD and to delineate effective strategies aimed at mitigating this economic burden. The key themes were:

#### Healthcare access and quality

The panelists reached consensus on the importance of ensuring equitable access to quality healthcare services as a means of addressing the economic burden of NCD. They emphasized that shifting from tertiary care to preventive strategies at the national level would be both cost-saving and health-promoting. The discussion highlighted the need for robust policy advocacy to address systemic challenges in Pakistan’s primary healthcare system, including inadequate infrastructure, limited medication availability, and workforce shortages. Although policies to encourage rural medical practice exist, challenges remain in ensuring accountability and adequate doctor time in rural facilities. The reluctance of doctors to work in rural areas, driven by low salaries, inadequate facilities, and poor living conditions, was cited as a major impediment to equitable service delivery.

#### Intersectoral collaboration

Panelists emphasized the need for intersectoral collaborations and policy interventions, such as tobacco taxation and alcohol regulation, to address NCD. Key points included increasing healthcare financing, reducing out-of-pocket payments, and implementing social security schemes to ease the economic burden.

#### Cost-effectiveness of healthcare interventions

Panelists emphasized the importance of evaluating the cost-effectiveness of preventive and care delivery interventions, particularly in rural and underserved areas. Data from initiatives such as satellite centers established by national institutes demonstrate the potential for both improved health outcomes and cost savings. Such evidence can be leveraged to advocate for policy reforms and to support the expansion of successful models across regions.

### Cancer prevention: is that an achievable goal in Pakistan?

Data from the National Cancer Registry of Pakistan highlights breast cancer as the leading cancer among females and oral cancer among males, indicating a severe public health crisis [7]. Within this context, the discussion aimed to identify priority areas for cancer prevention and explore avenues for collaborative policy development. The key themes that emerged were:

#### Comprehensive cancer prevention approach

Panelists emphasized the need for a holistic, multisectoral cancer prevention strategy that integrates stakeholders across health, policy, and community domains. The active involvement of the public in advocacy and policy development was also highlighted as critical to ensuring that community needs and priorities inform programmatic interventions.

#### Early detection and screening

The critical importance of early detection and organized screening programs was underscored, particularly for breast, cervical, and colorectal cancers. Screening represents a cornerstone of cancer prevention; however, Pakistan faces significant gaps, including limited program coverage and low participation in follow-up testing. Consequently, many cases are detected at advanced stages, limiting the effectiveness of treatment and survival outcomes.

#### Socio-economic and cultural factors

Socio-economic and cultural factors were identified as major impediments to effective cancer prevention and control. Social stigma, entrenched traditional beliefs, and the high costs of screening and diagnostic procedures deter timely health-seeking behavior. For women, discomfort in discussing reproductive and cancer-related health issues further complicates access to services, often leading to delayed diagnoses.

#### Community engagement and awareness

Community engagement was considered central to promoting cancer prevention. Leveraging local leaders, celebrities, and social media platforms was identified as a powerful strategy for disseminating information and fostering public support. Sharing personal narratives and incorporating public perspectives into advocacy were emphasized as effective means of enhancing program legitimacy and influencing government action.

#### Need for specialized cancer services

Panelists stressed the urgent need for dedicated oncology centers to optimize outcomes and improve survival rates. Expanding access to basic screening, strengthening referral systems, and prioritizing specialized oncological expertise within healthcare systems were identified as critical steps for improving cancer care in Pakistan. Cancer registries were viewed as potentially valuable for tracking disease trends and informing policy; however, Pakistan currently lacks unified cancer registries and standardized indicators for monitoring cancer risk factors and outcomes.

### Promoting mental health as a critical component of overall well-being

Approximately 20 million people in Pakistan experience mental health issues [8]. Despite this, there are only about 500 psychologists and 400 psychiatrists in the country [9]. The objectives of this panel discussion were to examine the determinants of mental health, explore evidence-based approaches and policies for prevention and control of mental health issues in the country.

#### Expanding the workforce through task shifting

Given the scarcity of specialists, panelists advocated “task shifting” approaches that empower non-specialist providers, general practitioners, nurses, and community health workers with basic mental health skills. Such models, already piloted in programs like *Thinking Healthy*, demonstrate cost-effectiveness and scalability in low-resource contexts.

Since most health consultations in Pakistan take place in private facilities, the absence of mental health integration into private practice represents a major gap. Training general practitioners in basic screening, referral, and counseling skills was identified as a pragmatic entry point to rapidly expand mental health services, particularly in urban areas where private care dominates.

#### Structural and systemic barriers

Panelists emphasized that fragmented financing, low budget allocations, and affordability challenges limit access to care. These barriers are compounded by stigma and cultural taboos that deter help-seeking. Without systemic reforms, including insurance coverage, social protection, and culturally sensitive awareness campaigns, mental health services will remain inaccessible for most Pakistanis.

#### Indigenous and innovative approaches

Discussions highlighted the potential role of culturally embedded actors, such as religious and spiritual healers, who are often first consulted for psychological distress. Rather than dismissing these actors, panelists suggested structured partnerships where they serve as referral points into formal care. Such integration may reduce stigma and expand outreach, particularly in rural and conservative communities.

#### Absence of national mental health policy

Without a unifying policy framework, mental health remains fragmented, donor-driven, and underfunded. Panelists emphasized that a comprehensive, context-sensitive policy is necessary to coordinate interventions, define referral pathways, and align service delivery with population needs.

### Sustainable urban design for a healthy environment

As Pakistan’s cities expand rapidly, they face a convergence of public health and environmental challenges. This discussion explored how sustainable urban planning can contribute to healthier urban living. The major themes were:

#### Urban density and health infrastructure

Panelists highlighted that extremely high settlement densities, currently reaching ~ 3,000 people per hectare in some cities, are unsustainable. High density without adequate services exacerbates risks of spreading communicable diseases, poor air quality, and limited access to care. They argued for evidence-based density thresholds, integrated primary health facilities, and urban designs that prioritize walking and cycling infrastructure.

#### Climate-responsive housing and materials

Heatwaves, increasing frequently due to climate change, were highlighted as a major urban health risk. Poor housing materials and inadequate ventilation amplify respiratory and cardiovascular vulnerabilities. Panelists called for building regulations that ensure affordable insulation, natural light, and green ventilation corridors, especially in informal settlements.

#### Green spaces as health infrastructure

Green spaces were framed not as “aesthetic additions” but as essential health-promoting infrastructure. Drawing on international benchmarks like the “3–30–300” rule, panelists recommended ambitious greening targets, particularly in Karachi, where green cover has fallen by nearly 40% over two decades. Increasing tree cover and accessible parks was linked to reduced heat stress, improved mental health, and lower reliance on cars.

#### Social determinants and inclusivity

Panelists emphasized that urban health cannot be separated from social equity. Planning strategies designed for affluent neighborhoods are often unsuitable for low-income communities such as Sohrab Goth. Inclusive planning requires engaging residents in design processes, ensuring linguistic and cultural diversity, and integrating marginalized groups into decision-making forums.

#### Governance and power dynamics

A recurring theme was the dominance of vested interests, developers, bureaucrats, and political elites in urban planning, often at the expense of community needs. The lack of zoning laws for walkways, pedestrian safety, or equitable housing reflects these imbalances. Panelists emphasized that transparent governance, stronger civil society advocacy, and multidisciplinary engagement are prerequisites for sustainable and healthy urban design.

## Discussion

The AKUPI-NCD symposium, held annually for three consecutive years, has become a significant platform for addressing NCD in Pakistan. These symposiums have provided a forum for scientists, policymakers, and stakeholders to share scientific evidence and discuss gaps in policy and research, contributing to a more coordinated NCD response. While similar conferences globally focus on abstract publications and policy recommendations, the AKUPI-NCD symposium took an innovative approach by involving policy and subject experts in panel discussions to generate actionable insights. This format allows for the development of bold recommendations that attract attention from both the government and key stakeholders, facilitate the creation of effective strategies to combat NCD.

This study synthesized discussions from over 30 national and international experts into a set of cohesive, context-specific recommendations. The value of this symposium lies not in identifying entirely new problems such as health system fragmentation, financial barriers, and the social determinants of health but in achieving a multi-sectoral expert consensus on their relative priority and on pragmatic, actionable solutions for the Pakistani context.

A key consensus was the need for a paradigm shift from a tertiary-care to a primary-care focus on a life-course approach, empowerment of individuals and communities, evidence-based strategies, and multisectoral collaboration. Similar recommendations were also highlighted in a conference held in Tanzania [8]. The WHO's Global Action Plan for the Prevention and Control of NCDs 2023–2030 highlights the life-course approach and strengthening primary healthcare as foundational pillars [9, 10]. This is particularly relevant in South Asia, as emphasized by the *Lancet* series on reorienting health systems in the region [11], which draws on experiences from Pakistan and its neighbors to argue that robust primary care is the only sustainable solution to the NCD crisis. Our findings on task shifting and integrating CVD in maternal health initiatives resonate strongly with studies advocating for integrated, life-cycle approaches to NCD prevention [12, 13]. Panelists emphasized that this shift is not merely clinical but requires political will to reallocate resources and establish dedicated NCD units within the government infrastructure.

The repeated emphasis on multi-sectoral action (e.g., involving urban planners for healthy cities and finance ministries for sin taxes) echoes successful strategies from other countries. Thailand's success in NCD control is widely attributed to its "Health in All Policies" approach and strong multi-sectoral collaboration, backed by strategic laws and a dedicated health promotion fund financed by sin taxes [14]. Similarly, Sri Lanka's recently launched National Multisectoral Action Plan 2023–2027 explicitly engages sectors beyond health, including education, sports, and urban development, mirroring our panelists' calls for a whole-of-government response [15]. The recommendation to reframe prevention as "health promotion" provides a strategic communication tool to increase public and political buy-in, addressing the lack of demand for preventive services, a challenge reported by a study [16].

The concrete proposal to mandate the '3–30–300' rule for urban greening provides a measurable, evidence-based target for urban planners, a level of specificity often missing from health policy recommendations. Furthermore, the suggestion to formally engage religious and spiritual healers as structured referral points for mental health care is a culturally intelligent strategy. This approach acknowledges the existing care-seeking pathways in Pakistan [17]. Similar models have been implemented in other contexts where traditional and modern health systems coexist, though this approach requires careful implementation to ensure both ethical and effective collaboration [18].

However, these recommendations face significant implementation barriers. A major constraint is the limited government health budget, which prioritizes acute care over prevention. Implementing nationwide screening or establishing cancer registries competes with other urgent health priorities. Furthermore, weak primary healthcare infrastructure and human resource shortages, particularly in rural areas, pose a fundamental challenge to shifting care downstream. The suggested task-shifting in NCD and mental healthcare, while necessary, requires sustained investment in training and supervision to be effective and safe.

The symposium addresses multiple critical aspects of NCD in Pakistan, including cardiovascular health, cancer, mental health, and urban design, offering a holistic perspective. By bringing together policymakers and public health experts, the event ensured diverse viewpoints and facilitated collaborative dialogue, enhancing the potential for impactful recommendations. However, a critical limitation of this paper is that the perspectives are exclusively from experts and policymakers. The absence of community members and end-users means the recommendations, while technically sound, may not fully account for practical acceptability and feasibility on the ground. Future research must incorporate these voices to ensure recommendations are people-centered and equitable.

## Conclusion

This symposium successfully synthesized expert consensus into a prioritized agenda for NCD prevention and control in Pakistan. The findings highlight that tackling the rising NCD burden requires more than medical interventions; it demands a holistic, multi-sectoral approach that addresses the underlying social, economic, and environmental determinants of health. While the challenges are significant, the clear, context-specific recommendations provide a roadmap for action. The next step is to translate these evidence-informed insights into policies with allocated resources and robust monitoring and evaluation frameworks.

## Supplementary Information

Supplementary Material 1.
