# Supplementary material for: Non-communicable diseases prevention and control in Pakistan: recommendations from policy and public health experts
Source: BMC Proc. 2025 Oct 14;19(Suppl 30):32. doi: 10.1186/s12919-025-00350-4 (PMC12519595; doi:10.1186/s12919-025-00350-4)
Supplement: Supplementary file 1 — Supplementary Material 1. [file 12919_2025_350_MOESM1_ESM.docx]

**Supplementary File 1: Direct Quotes from Panelists**

1. **Cardiovascular Health in Pakistan: Shaping Policy for Primary Prevention**
   1. **Lack of successful models in Pakistan**

*“Health departments don't have dedicated infrastructure and staff for NCD”.*

*“Enough infrastructure, awareness, and human resources are in cities, we need people with power and pen to sit in the federal and provincial ministers to have this intersectoral liaison to develop national level programs”.*

- 1. **Reframing CVD prevention and utilization of media**

*“Promoting health is a real way to frame this. Let's talk about improving your quality of life, energy and your stamina, being alive longer so that you can see your children and perhaps your grandchildren grow up and do that healthily so that you can engage”.*

*“Utilizing media and social media, is the call of the day, which is something people are now listening rather than attending lectures, seminars, conferences, capacity building”*

- 1. **Tailoring communication strategies**

*“Learn the languages of those whom we are addressing to, when we are addressing patients, speak in terms they understand just as we must engage with communities in their language. Similarly, when engaging policymakers, it's important to tailor our message to their interests and priorities”.*

- 1. **Leadership from health institutions**

*“The community sees non-communicable diseases as unavoidable, so it's our job as a health institution to create awareness and drive demand. Even with limited government support due to funding constraints, we can take the lead and inspire collaboration by expanding our efforts and conducting more research in this area”*

*“Engaging with policymakers from the start ensures our research aligns with their needs and timelines, making our findings more impactful”.*

- 1. **Holistic approach and continuum of care: starting from intrauterine**

*“The best time to focus is when the baby is intrauterine”.*

*“Preventative medicine starts when the baby is intrauterine. That is where we are not concentrating at all. When we see somebody at the age of 20 and 30 and say you are, you have got obesity, or you may have a heart attack. We are already too late, too late”.*

*“The other side you look into other than the health and the poverty, gender imbalance is in the sector (health). So, the problem is complex, one policy will not work”*

- 1. **Public private partnership: shift from tertiary to primary prevention**

*“I believe in the role of public private partnerships because I'm a private implementer and I know that we can't do a lot particularly expansion and scale is not possible until you partner with the public health institutions such as the government or the hospital working in the area and. Our scale only comes by partnering with them.”*

*“The only way you can reverse the wheel is to start from primary healthcare, build the capacity of LHWs, midwives and medical doctors working at community level.”*

- 1. **Data interoperability: essential for continuity of care**

*“If primary care is not connected to tertiary care units, you cannot create continuity of care at all levels”.*

1. **Economic Perspectives on Cardiovascular Disease Prevention Navigating Contextual Challenges**
   1. **Healthcare access and quality**

"To lower the economic burden, we're going to have to think about how we can shift things from acute care to more preventive care."

- 1. **Intersectoral collaboration**

"Let's not underestimate the importance of intersectoral interventions. policies like tobacco taxation, alcohol taxation”.

"Before we go into intersectoral collaborations and so on I think we just also have to look inside and know the silos that these people are working in."

- 1. **Policy advocacy**

"We need to come together as academics, as researchers and advocates, and have a serious conversation with policymakers at the provincial and federal level."

"The main agenda is drinking water facility, provision of drinking water, malnutrition screening, infectious Disease Control, EPI program and the vaccination of children and mother and child health antenatal and perinatal care."

- 1. **Cost-effectiveness of healthcare interventions**

"We should look at costs, but in this case, you also look at quality. I think that's critical for any intervention of that nature."

“You need to look at increasing healthcare finances. Social Security has a big role to play, particularly when people have already suffered the consequences."

1. **Cancer Prevention: Is That an Achievable Goal in Pakistan?**
   1. **Comprehensive cancer prevention approach**

*"It requires a lot of comprehensive and coordinated efforts in incorporating various sectors across the spectrum including healthcare professionals, policymakers, governmental agencies, and the community itself."*

*"The government response is driven by a lot of public emotion and other factors. Including the public in the equation when engaging in advocacy can reap required results."*

- 1. **Early detection and screening**

*"Screening mammography rate is very low even among educated people."*

*"Early stages are game changer."*

*"Institute make screening mammography mandatory for their female staff because we are seeing even among colleagues people coming with third and fourth stage."*

*3.3* **Socio-economic and cultural factors:** **hindrances to effective cancer prevention and control**

*"Many women don't go back for additional test or biopsies. These are areas that need to be addressed."*

*“Many women don't go back for additional tests or biopsy. You know, again, these are areas that need to be addressed. And there are many barriers, stigma, embarrassment, but the most important is, what it could be? Cost: it's very expensive, right? Even when you are offering subsidized additional tests, ultrasound, you know, it makes it very difficult and anxiety and there are many other factors. So we have to address these things if we want to increase this screening mammography. And this screening mammography is also important to reach to underserved women.”*

- 1. **Healthcare infrastructure and policy**

*"We don't have any palliative services for them... It's a very neglected area."*

*"There's no unified registry of cancers... We need to have an integrated strategy."*

- 1. **Community engagement and awareness**

*“When we talk about the public awareness campaigns, we shy away from contextualizing the things in the local context. So, the messages should be sensitive to the public.”*

*“Here it is very essential to mention that in the realm of public policy, the government response is driven by a lot of public emotion and other factors. The legislature tends to respond to it because of the vote bank. Therefore, including the public in equation when engaging in advocacy can reap required results. Therefore, public opinion-making should be a factor when we approach cancer prevention policy. That is at the very outset of things.”*

- 1. **Need for Specialized Cancer Services**

*"We have to have dedicated service... Instead of they are being treated by general specialists."*

*"Our government has to develop some system... Where to go if anything is coming up."*

1. **Promoting mental health as a critical component of overall wellbeing**
   1. **Recognition of mental health as integral to overall health**

"I'm very pleased that mental health is included in this discussion to start with because mental health often comes as an afterthought in our conversations about public health in Pakistan."

"If we do not include mental health, then our understanding of health is incomplete."

"As the burden of physical illnesses grows, so does the burden of mental health issues."

"We tend to ignore mental health, and when you ignore something, it continues to grow."

- 1. **Broadening mental health training beyond specialists**

"The discussion shouldn't revolve solely around psychiatrists or psychologists. Mental health is too vital to be left to specialists alone."

"We should train all healthcare professionals, starting from community health workers upwards, to address mental health issues effectively."

"Task shifting is crucial. We need to demystify mental health and spread training across all health professionals, from nurses to paramedics."

- 1. **Challenges and opportunities in the private sector**

"Approximately 70% of healthcare consultations in Pakistan occur in the private sector, presenting a significant challenge."

"GPs in private practice often lack training in mental health. Addressing mental health in the private sector requires tailored interventions."

"The scarcity of psychiatrists in private hospitals is a concern. Specialized training programs for GPs in mental health are essential."

- 1. **Systemic challenges and solutions**

"There's a lack of prioritization of healthcare in our budget. Real change requires a commitment to addressing broader issues and implementing structural reforms."

"Short-term interventions are necessary, but systemic challenges need addressing. Affordability, availability, accessibility, and cultural pertinence are critical."

"We need to completely overhaul the design of healthcare delivery. A systemic approach is necessary, addressing health promotion, system development, curriculum, workforce, monitoring, evaluation, and innovation."

- 1. **Innovative approaches and indigenous solutions**

"There are indigenous solutions. Traditional and spiritual healers can serve as gatekeepers for the first episode of psychosis."

"Task-sharing interventions like the Thinking Healthy program have proven effective in low-middle-income settings. Scaling up such programs is crucial."

"Indigenous solutions can be explored, and traditional healers can play a role in referral points. Activism and advocacy for mental health are needed to grab people's attention."

- 1. **Absence of national mental health policy**

"Firstly, it's imperative to note that Pakistan lacks a national mental health policy. We need a comprehensive policy tailored to our context, outlining appropriate interventions."

"A comprehensive mental health policy is the foundation we're missing. It should be designed for our unique situation, not an afterthought in a document dominated by other health concerns."

"Without a dedicated mental health policy, the issue remains marginalized. It needs to stand on its own, not buried in a larger health document."

1. **Sustainable Urban Design for A Healthy Environment**
   1. **Density of settlements**

“Right now, we have settlements that are 3000 persons per hectare and that too in at night. You cannot have healthy settlements with such densities. It is not possible.”

**5.2 Light and ventilation and building structure material**

*“Light and ventilation you can guarantee but insulation require for low income settlements. Insulation requires a lot of research because we do not have cheap means of insulation, but I think we can develop them with a few pilot projects.”*

“*Right now, I am trying to develop one and hopefully we can reduce cost to a limit to a level where low income communities can afford them*.”

“Healthy urbanisms: A concept developed by the World Health Organization in 1980s. Cities like China have healthy building standards, based on which they also focus on physical and mental well-being and health, and on the fact that there should be leisure spaces for people of all ages.”

*“We have to think in scales of different kinds of social, cultural context, the kind of limitations that cultures, societies, ethnicities, and languages place on us as very disaggregated kinds of citizens. It's very important to start thinking in terms of pockets of populations, which means that we scale it down, disaggregate, and understand it in terms of particular needs for particular places and particular people.”*

*“I'm building a hospital in Quetta and one thing I have done is in the middle of the building I put huge ramps from basement to the top floor in addition to the elevators as well as stairs. We should not be dependent on electrical means. Being in Pakistan, I don't want my patient to be stuck in the elevator in in case of the power outage. And when you when you are providing these type of spaces, ample natural light green spaces, you are encouraging not only your employees to walk but also your patient.”*

**5.3 Necessary portion of green spaces**

“Countries already applying this principle are now aiming for 50% tree cover and 500-meter walkable distance to green spaces.”

**5.4 Understanding of disease and its causes**

*“Understanding of disease and its causes, so that you can work to remove those causes in social and physical planning. And here I give reference to the Moscow Master Plan 2020, which was all about serving what diseases existed and what were their causes and how those causes could be removed.”*

- 1. **Prioritize assistance over invention**

“We don't necessarily need to reinvent the wheel. We can look at the kind of practices that are working for them and then propose how they can be made better.”

- 1. **Cultural inclusivity**

*“I think the problem basically is that you have other interest groups apart from those who consider themselves to be planners and promoters of community interests. That is usually a very powerful developer, bureaucrat, politician, Nexus. And I think this Nexus is really your planner. How do you change this under law every 10 years you have to have a so-called master plan. Now in my experience the real communities never get a chance to participate in the planning process.”*

*“We should be culturally sensitive and inclusive. So, whatever you plan in Sohrab Goth, considering the religious tendency, you cannot do the same in highly affluent areas. I think just as a physician we listen to our patient, we should listen to the community, we should engage them through workshop, through education, we should utilize digital platforms, we should conduct meetings, we should form some liaison team that includes the policy makers, the designers, the developers as well as the end users.”*

*“People are suffering, you have removed the air corridors, right? Did we ask those people when we were designing it, 7 out of 24 Chest pain units in Karachi are under the bridges and we have seen first hand the impact of that.”*

- 1. **Conflict of interest while making policy**

“When we raise the question that we've never really created any master plans from a health perspective, there's the Karachi city plan for 2047 that's about to come. I don't know if any health expert has been included in that master plan or has any been included for the same climate change policy that is under works.”
